# Electroacupuncture for Motor Recovery in Subacute Stroke: A Randomized Trial Exploring CReST‐Related Modulation

**DOI:** 10.1002/brb3.71496

**Published:** 2026-05-19

**Authors:** Nan Xia, Qiongfang Wu, Tangzhu Yang, Chaoyuan Liang, Zhenhong Li, Zejian Chen, Minghui Gu, Jinghong Li, Mingzhu Wang, Yong Chen, Xiaohua Han

**Affiliations:** ^1^ Department of Rehabilitation Medicine, Tongji Hospital, Tongji Medical College Huazhong University of Science and Technology Wuhan China; ^2^ Department of Rehabilitation Medicine Wuhan No.1 Hospital Wuhan China; ^3^ Rehabilitation Medicine Department of Wuhan University of Science and Technology Affiliated Wuchang Hospital Wuhan China; ^4^ Department of Electrical and Electronic Engineering University of Manchester Manchester UK

**Keywords:** corticoreticulospinal tract, electroacupuncture, sensorimotor network, stroke rehabilitation

## Abstract

**Background:**

Upper limb impairment is a common consequence of stroke, with unclear motor recovery mechanisms. Electroacupuncture (EA) has shown promise in promoting recovery, but its effects on the corticoreticulospinal tract (CReST), a key stroke rehabilitation pathway, remain underexplored. This study aimed to investigate the effects of EA on motor recovery and its potential modulation of CReST‐related pathways in subacute stroke patients.

**Methods:**

Thirty‐two subacute stroke patients were randomly assigned to the EA or control group. Both groups underwent a two‐week rehabilitation program, with the EA group receiving EA over the Hegu (LI4) and Quchi (LI11) acupoints before physical therapy. The Fugl‐Meyer Assessment for Upper Extremity (FMA‐UE) and the Modified Ashworth Scale (MAS) assessed motor function and spasticity before and after the intervention. Surface electromyography and functional near‐infrared spectroscopy (fNIRS) measured motor performance and neural activity.

**Results:**

The EA group exhibited significant improvements in FMA‐UE scores and faster premotor reaction times compared to controls. Increased StartReact incidence and altered anticipatory muscle activations were observed in the EA group. fNIRS analysis revealed an enhanced contralateral sensorimotor network (SMN) activity during the early stage of EA intervention. Additionally, more severe motor impairments were associated with greater contralesional SMN connectivity improvement during the early EA phase.

**Conclusions:**

Preliminary evidence suggests that EA at LI4 and LI11 serves as a promising adjuvant intervention to enhance upper limb motor recovery in subacute stroke; this benefit is likely mediated by the modulation of CReST‐related pathway and contralesional SMN reorganization. Larger, well‐controlled studies incorporating sham‐EA groups are warranted to validate these findings and establish definitive causal links.

## Graphical abstract

This diagram illustrates the potential role of Electroacupuncture (EA) as a neuromodulatory primer for enhancing upper limb motor recovery via CReST‐related pathways in subacute stroke.Potential Contralesional Modulation (Blue Panels): Ascending sensory inputs from EA at LI4 and LI11 are associated with an acute enhancement of functional connectivity within the contralesional sensorimotor network (SMN). This cortical response appears to be time‐sensitive, with the most pronounced connectivity changes observed during the initial 10 minutes of stimulation.Suggested Descending Recruitment (Red Panels): The transient increase in contralesional SMN activity may facilitate descending signaling along the ipsilaterally‐projecting CReST. This recruitment is indirectly reflected by electrophysiological markers, including an increased StartReact (SCM+) incidence, altered muscle activation patterns, and shortened premotor reaction times (PRT) in the affected limb.**Clinical Observations**: Integration of EA prior to physical therapy (PT) appears to optimize the neural foundation for subsequent training, which may contribute to the significant improvements observed in Fugl‐Meyer Assessment (FMA‐UE) scores.

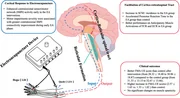

## 1 Introduction

Stroke, a leading non‐communicable disease with profound impacts on global health, remains a critical focus of medical research and intervention (GBD 2019 Stroke Collaborators 2021; Stinear et al. 2020). In mainland China, the burden of stroke is particularly heavy. As of 2020, the estimated overall prevalence among individuals aged 40 years and older reached 2.6%, with an incidence rate of 505.2 per 100,000 person‐years and a mortality rate of 343.4 per 100,000 person‐years (Tu et al. 2023). Notably, while prevalence is higher in urban settings, rural areas face disproportionately higher incidence and mortality rates. This disparity is often exacerbated by the scarcity of affordable and accessible rehabilitation technologies in resource‐limited regions, highlighting a critical need for cost‐effective interventions that can be widely implemented. Upper extremity motor dysfunction is a common and debilitating outcome of stroke, often manifesting as loss of voluntary movement, impaired motor control, or involuntary muscle co‐activation. These motor impairments severely hinder essential daily activities, such as dressing, eating, and other self‐care tasks. Recent research reveals that only 11.6% of individuals achieve complete functional recovery of the arm, while 62% show no signs of dexterity recovery even six months post‐stroke (Kwakkel et al. 2003). These findings underscore the urgent need for innovative and effective rehabilitation strategies. Despite notable progress, there remain considerable gaps in our understanding of the underlying mechanisms of stroke‐related motor impairment and in identifying the most effective treatment modalities to bridge these recovery deficits (Saikaley et al. 2022; Stinear et al. 2020).

Electroacupuncture (EA), as a cost‐effective and minimally invasive therapy, offers a promising strategy to bridge this gap, especially in resource‐limited settings where resource‐intensive and technologically demanding rehabilitation modalities are less accessible. Specifically, EA at the acupoints Hegu (LI4) and Quchi (LI11) provides a unique therapeutic approach rooted in traditional Chinese meridian theory. According to this framework, upper extremity motor dysfunction post‐stroke is thought to arise from disrupted flow of “*qi”* and “*blood”* along the *Yangming meridian*. By applying controlled electrical currents to these acupoints, EA is believed to restore the flow of “*qi*” and “*blood”* in the hemiplegic limb, potentially promoting motor recovery. Preliminary clinical studies have suggested that EA may offer beneficial effects on motor outcomes for stroke survivors (Wu et al. 2010). A literature review highlighted acupuncture's potential to promote neurogenesis, cell proliferation, and regulate cerebral blood flow (Chavez et al. 2017). Moreover, EA may also modulate brain networks (Chen et al. 2023; Wang et al. 2023) involved in motor planning, sensory processing, and motor initiation. In addition, a recent electroencephalogram study reported a positive response in the delta band power during EA at the above two acupoints (Zou et al. 2020), suggesting that EA may play a key role in facilitating neuroplasticity after stroke.

Another area of interest in stroke rehabilitation is the role of the corticoreticulospinal tract (CReST). Following impairment of the corticospinal tract (CST), the CReST may serve as a critical survival‐oriented neural adaptation to maximize motor output. Changes in CReST function are closely associated with the development of spasticity (Li et al. 2017), stereotyped muscle synergy patterns (Senesh et al. 2020), and anticipatory postural adjustments (Xia et al. 2021) following stroke. Furthermore, activation of the reticular nucleus has been shown to correlate positively with maximal muscle contraction (Danielson et al. 2024). However, how these changes can be harnessed to enhance descending motor control or prevent maladaptive movement patterns post‐stroke remains poorly understood (Mooney et al. 2024). Preliminary evidence suggests that acupuncture may influence the excitability of spinal motor neurons and when combined with exercise training, it could improve muscle spasticity post‐stroke (Zhu et al. 2019). We hypothesize that EA may modulate CReST plasticity, potentially facilitating motor recovery by optimizing the recruitment of descending motor pathways.

This randomized controlled pilot trial aims to investigate the potential relationship between EA intervention and changes in CReST performance, laying the groundwork for subsequent mechanistic studies and clinical applications. With the advent of advanced technologies, we can now assess the effects of EA on stroke recovery with greater precision. Building upon previous research, we will employ the “StartReact Effect (SE)” test, which utilizes muscle activation responses under different excitation states—acoustic startle or non‐startle conditions—to reflect changes in CReST function (Xia et al. 2021). The proportion of SE occurrences during tasks (DeLuca et al. 2022), along with the latency of agonist initiation (Choudhury et al. 2019) and anticipatory muscle activations (AMAs) (Xia et al. 2021), are increasingly used to evaluate CReST function in stroke patients. Based on these findings, we anticipate that this algorithm can be applied to the current study of EA for upper limb intervention, offering insights into CReST modulation that may not be captured by conventional assessments.

Moreover, after a stroke, the nervous system often experiences “disconnection” between neural networks (Catani and ffytche 2005). Addressing how to promote information transmission across these disconnected networks and enhance neural co‐firing has become a key therapeutic goal (Ganguly et al. 2022). Extensive neuroplastic reorganization has been reported in both the ipsilateral and contralateral sides of the lesion in post‐stroke patients (Di Pino et al. 2014). Imaging studies have indicated that CReST involves widespread activation across the frontal and parietal cortices (Boyne et al. 2021), providing a structural foundation for the reconstruction of neural connections. Given that changes in cortical activity in isolated regions may not fully represent the complexity of brain network reorganization, it may be limiting to focus solely on changes in the activation of individual cortical areas (Grefkes and Fink 2011). Therefore, to capture the dynamic alterations across the entire frontal and parietal networks during EA intervention, we plan to utilize functional near‐infrared spectroscopy (fNIRS), with a focus on functional connectivity (FC).

These outcomes, derived from surface electromyography (sEMG) and fNIRS, will provide objective, real‐time data on how EA influences cortical response, CReST function, and motor performance in stroke populations. By providing a comprehensive view of EA's impact on frontoparietal modulation and CReST remodeling, this study aims to contribute preliminary evidence for EA's therapeutic potential and offer deeper insights into its role in stroke rehabilitation.

## 2 Materials and Methods

### 2.1 Participants

**FIGURE 1:**
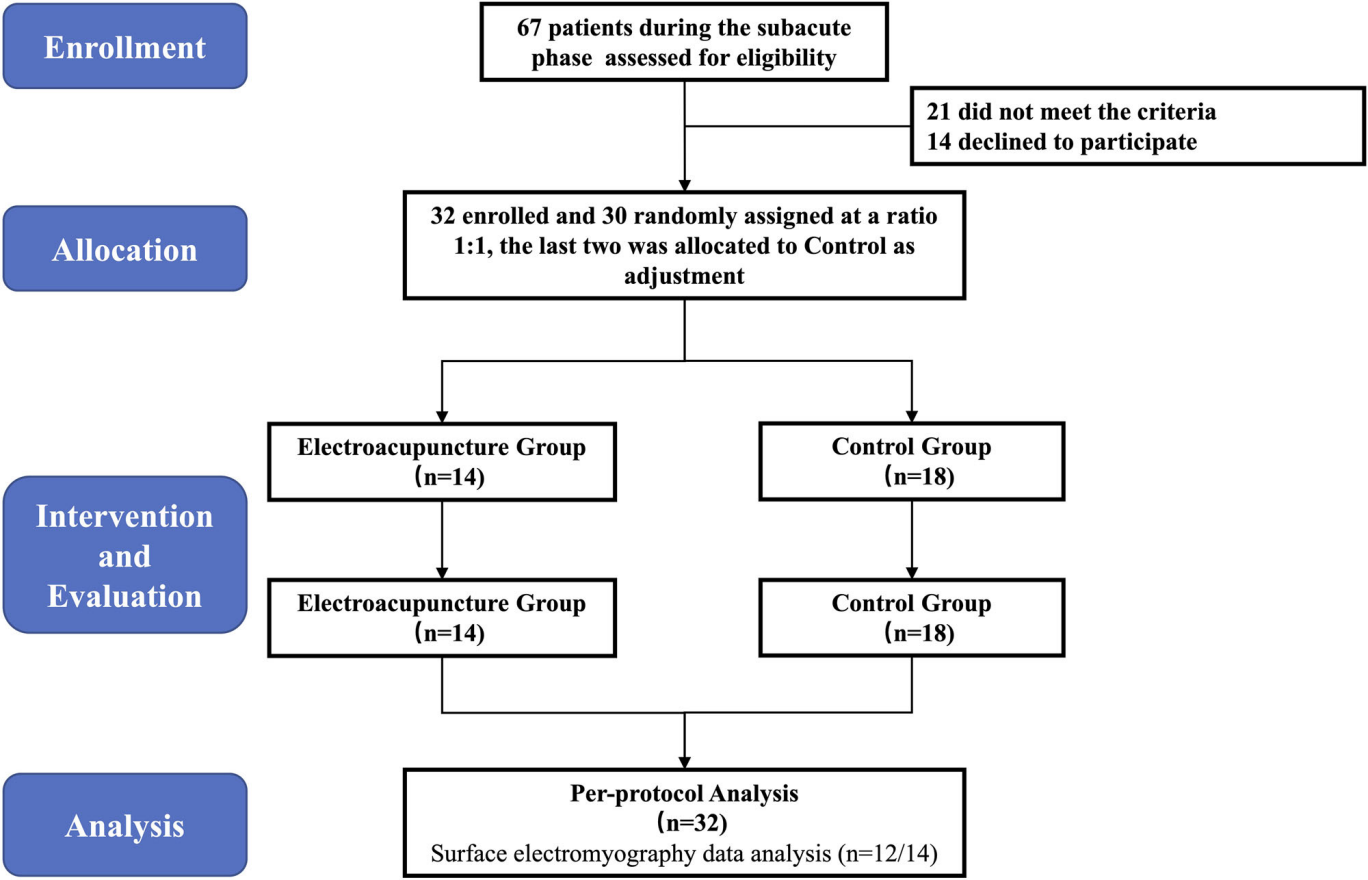
**The flow diagram of the present study**.

Thirty‐two stroke patients were recruited between December 2022 and June 2024. Sample size was estimated using G*Power to achieve 80% power for detecting an effect size (Cohen's *d*) of 1.1 and adjusted to 32 for potential dropouts. Participants were randomly assigned (block size = 4) by computer‐generated sequence. Outcome assessors and analysts were blinded. Figure 1 shows the study flow. **Figure** **1** provided the study flow chart.

Inclusion criteria: age 18–70, first‐ever ischemic/hemorrhagic stroke (subacute phase) with unilateral motor deficits and ≥30° active shoulder flexion. Exclusion criteria: brainstem lesions, epilepsy, severe spasticity, frozen shoulder, fractures, electronic implants, or fNIRS contraindications.

Motor function and spasticity were evaluated with the Fugl‐Meyer Assessment (FMA‐UE) and Modified Ashworth Scale (MAS). Both groups received 2 weeks of standard rehabilitation (15 h/week). Participants in the experimental group additionally underwent a daily 20‐min electroacupuncture (EA) session immediately prior to their scheduled physical therapy. To maintain procedural consistency, the subsequent physical therapy was initiated within 15 min after the completion of EA.

### 2.2 Electro‐Acupuncture Protocol

**FIGURE 2:**
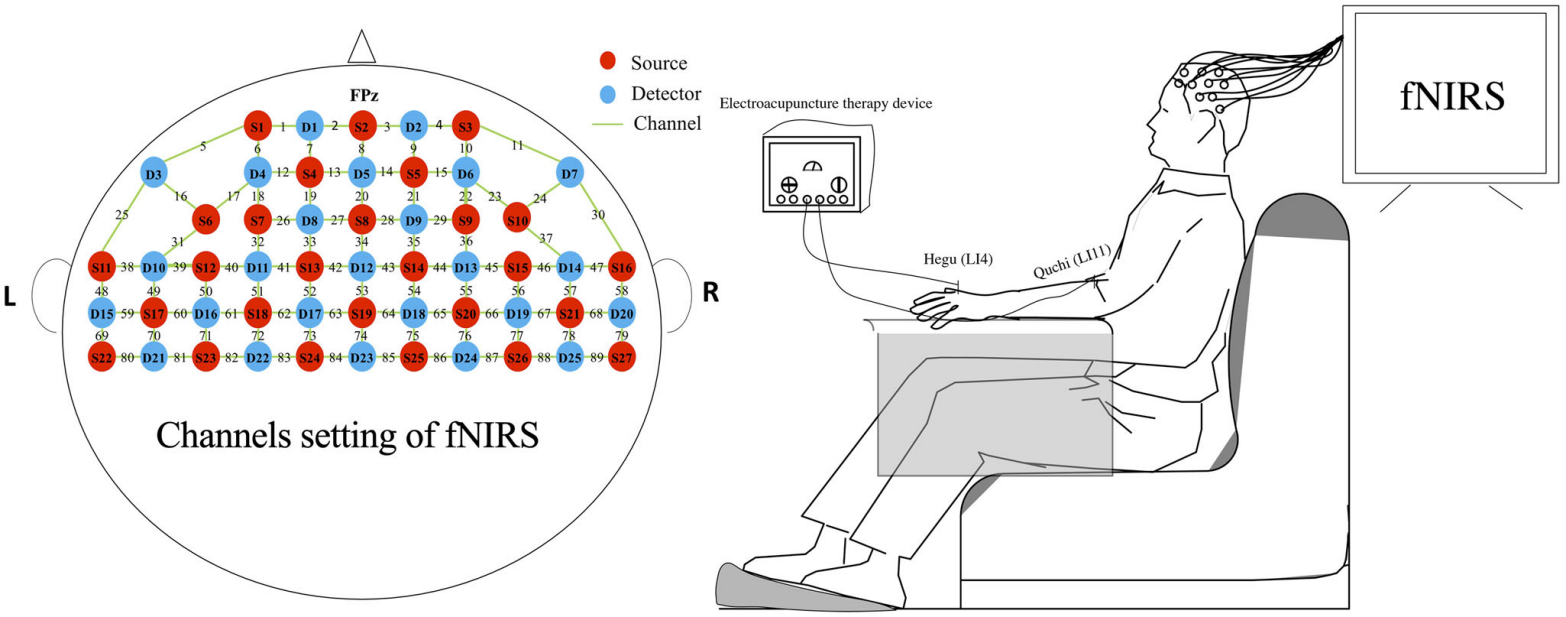
Schematic of EA intervention and fNIRS data collection setup This diagram illustrates the placement of acupuncture needles at the “Hegu” (LI4) and “Quchi” (LI11) acupoints, alongside the configuration of fNIRS sensors on the subject's head for real‐time monitoring of functional connectivity during the electroacupuncture (EA) intervention.

As shown in **Figure** **2**, EA was applied by a certified acupuncturist at LI4 (Hegu) and LI11 (Quchi) on the affected side. Sterile disposable needles (0.30 × 40 mm) were inserted vertically to a depth of 15–25 mm. After *Deqi* induction (characterized by the patient's subjective report of soreness, numbness, distension, or heaviness), the needles were connected to an EA stimulator (SDZ‐II, Suzhou, China) delivering 2 Hz intermittent waves for 20 min (Zou et al., 2020). The stimulation intensity was adjusted until a visible muscle twitch was elicited while remaining within the patient's comfort threshold. Following the 20‐minute stimulation, needles were removed, and the sites were pressed with sterile dry cotton balls for approximately 30 s to ensure hemostasis and prevent hematoma, after which patients were immediately transferred for physical therapy.

### 2.3 Surface EMG Analysis for Reaching Task

Participants performed a reaching task using the paralyzed side's palm or forearm to touch the center of a tripod tray, positioned at 80% shoulder height, 45° anterolaterally, and at a distance equal to the subject's arm length from the acromion, both before and 12 h after the 2‐week intervention (Xia et al. 2021). The task consisted of 30 trials with two auditory stimuli: 15 trials of a 40 ms beep at 80 dB for movement initiation and 15 trials of 40 ms white noise at 114 dB, presented in random order to reduce predictability. The white noise (114 dB) induced startle‐evoked (SE) responses (Valls‐Solé et al. 2005), characterized by early activation of bilateral sternocleidomastoid (SCM) muscles and contractions in agonist and stabilizer muscles (Marinovic and Tresilian 2016; Maslovat et al. 2015). SE serves as a marker of CReST plasticity, reflecting its role in rapid, coordinated motor responses (Carlsen and Maslovat 2019).

Surface EMG (Noraxon Ultimu, 2000 Hz) was recorded from the anterior deltoid (AD), flexor carpi radialis (FCR), extensor carpi radialis (ECR), and bilateral sternocleidomastoids (SCM) following SENIAM guidelines (Hermens et al. 1999). The sEMG data were processed using Psychtoolbox‐3 in MATLAB, with signals recorded by the Ultimus sEMG system (Noraxon USA Inc.) at a sampling rate of 2000 Hz.

### 2.4 Data Processing for StartReact Effect

sEMG data were processed in MATLAB. First, signals were segmented, notch filtered at 50 Hz, and bandpass filtered between 30 and 300 Hz. Onset time was determined by setting a threshold as the mean plus three standard deviations of the baseline (2500–500 ms prior to each auditory stimulus). Time zero (*T*_0_) was defined as the activation onset of AD.

Premotor reaction time (PRT) was calculated as the interval between the auditory “Go” cue and T0. Trials with PRT outside 30–400 ms were excluded. Muscle onset latency was defined as the time from activation onset to T_0_ in trials with positive AMAs (Xia et al. 2021).

For SE, a positive SCM response (SCM+) was identified if SCM activation occurred within 30–130 ms after the 114 dB stimulus, while no significant SCM activation was classified as negative SE (SCM‐). The key outcomes were positive SE incidence, AD PRT, and AMAs delay in ECR and FCR. Trials were categorized into SCM+, SCM‐, and normal sound‐primed trials, with the respective indicators calculated for each subject.

### 2.5 fNIRS Data Acquisition

fNIRS data was collected on days 5–7 of treatment during one randomly selected EA session using the BS‐7000 system (Wuhan Znion Technology Co., China). Data were recorded in three phases: baseline (6 min before EA), EA intervention (20 min), and post‐intervention (6 min after needle withdrawal). Segments were trimmed in MATLAB for uniformity and labeled as “pre,” “mid 1,” “mid 2,” and “post.”

The Montreal Neurological Institute coordinates and the corresponding Brodmann areas of each channel are provided in **Supplementary Material** **1**. A schematic diagram (**Figure** **2**) illustrates the experimental setup and procedure for EA and fNIRS data collection.

Preprocessing was conducted using the Homer 2 package in MATLAB, including conversion to optical density, motion artefact correction, bandpass filtering (0.01–0.1 Hz), and hemoglobin concentrations using the Beer‐Lambert law. For frequency‐domain analysis, amplitude spectra were derived using fast Fourier transform. Fractional Amplitude of Low‐Frequency Fluctuation (fALFF) was calculated as the ratio of amplitude in the 0.01–0.08 Hz range to the 0.01–0.25 Hz range.

FC within the frontoparietal (FPN) and sensorimotor (SMN) networks was assessed by Pearson correlation coefficients between time‐series data from all channel pairs. Intra‐hemispheric connections were analyzed by dividing the channels into left and right hemispheric groups. Stroke participants with left hemisphere lesions had their data flipped to align with the right‐brain‐dominated left‐arm intervention model. Data processing was performed using the NIRS‐KIT toolbox.

### 2.6 Statistical Analysis

Statistical analyses were performed using SPSS (version 26.0), with significance set at *p* < 0.05. Normality of numerical variables was assessed using the Shapiro–Wilk test, and results are presented as means ± standard deviations. Demographic data were compared using a chi‐square test for categorical variables and an independent samples *t*‐test for continuous variables. Changes in FMA‐UE scores were analyzed using ANCOVA. MAS scores and SCM+ incidence were compared using a non‐parametric rank‐sum test for within‐ and between‐group differences. Two‐way ANOVA with Bonferroni correction assessed the effects of trial type (SCM+, SCM‐, and normal) and group on PRT and AMA onset latency, with pairwise comparisons performed using independent samples *t*‐tests. For fNIRS data, one‐way repeated measures ANOVA examined changes across four time points (“pre,” “mid 1,” “mid 2,” and “post”), with Bonferroni‐corrected pairwise comparisons. Pearson correlation analysis was used to explore relationships between changes in brain network connectivity and significant indicators.

## 3 Results

Two participants in the control group had missing surface electromyography data post‐intervention due to skin allergies. To ensure comparability of primary outcomes, the last two enrolled participants were included. In total, 32 subacute stroke survivors participated (14 in the EA group, 18 in the control group). Valid sEMG data were obtained from 12 participants in the EA group and 14 in the control group for pre‐ and post‐intervention analysis. All participants completed the 2‐week rehabilitation program and FMA assessments, with the EA group also undergoing fNIRS testing. Demographic and clinical characteristics are shown in **Table** **1**.

**TABLE 1:** The demographic and clinical characteristics of the two groups.

Variable	EA group *n* = 14	Control group *n* = 18
**Age**	54.29 (7.74)	47.89 (12.48)
**Sex**, %female	50.00%	72.22%
**Time post stroke onset** (days)	52.43 (28.71)	57.56 (23.16)
**Stroke type**, %ischemic	64.29%	44.44%
**Lesion location**, (cortex/subcortex/cortex and subcortex)	1/9/4	4/12/2
**Paretic side**, %right	71.43%	61.11%
**Fugl‐Meyer Assessment for Upper Extremity score**	28.29 (18.40)	31.33 (13.15)
**Modified Ashworth Scale** (median (IQR))	1 (0,1)	1 (0,1)

Abbreviation: IQR, interquartile range.

### 3.1 Clinical Outcomes

**FIGURE 3:**
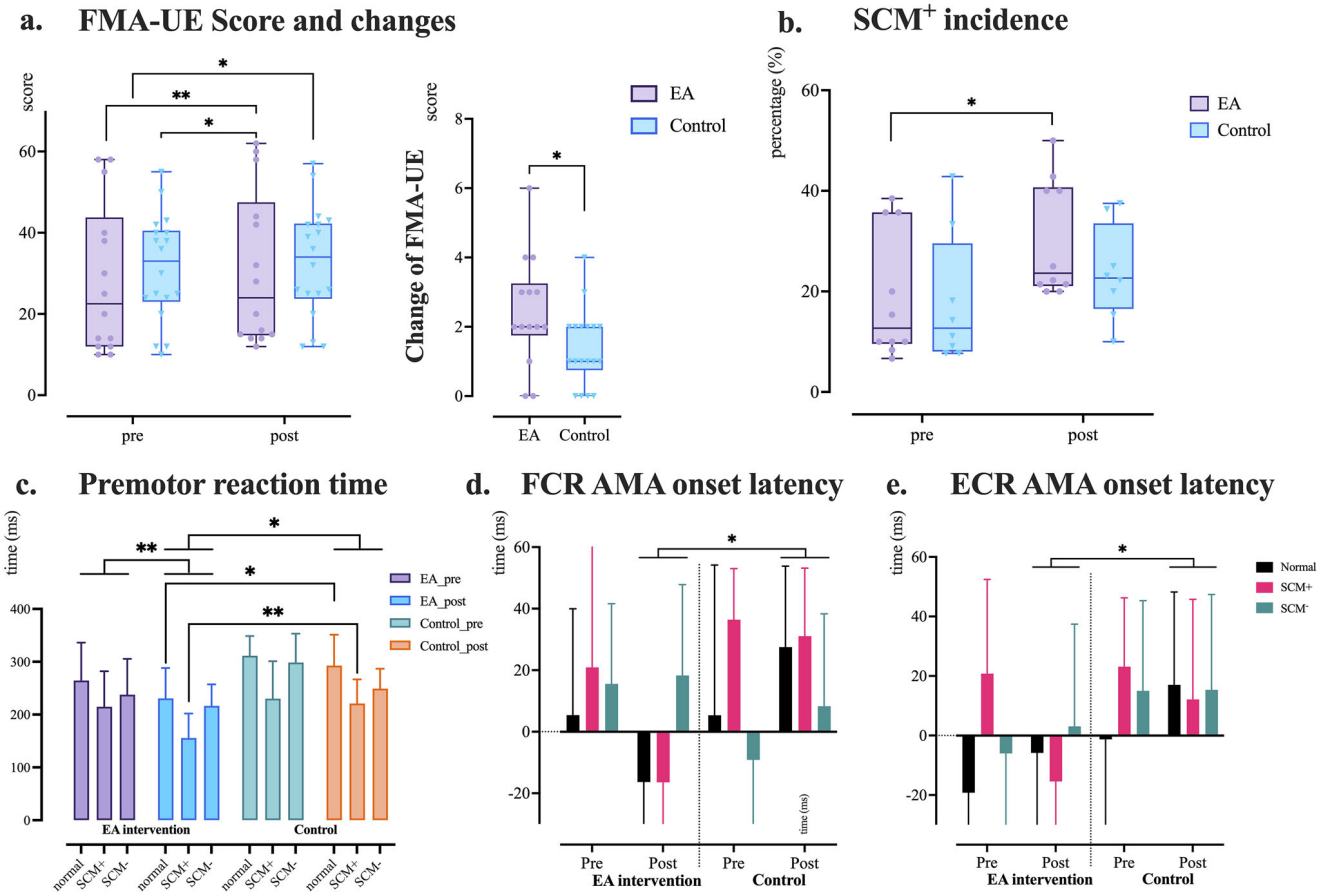
**Comparative analysis of motor function and corticoreticulospinal tract facilitation indicators between the two groups**. (a). Comparison of Fugl‐Meyer Assessment for Upper Extremity (FMA‐UE) scores and changes before and after intervention between the two groups, (b) Comparison of the incidence rate of premature activation of the sternocleidomastoid (SCM) muscle on either side in startle‐initiated trials between the two groups, (c) Comparative analysis of the starting reaction time of the deltoid muscle on the exercise side in normal, SCM+, and SCM‐ trials before and after the intervention between the two groups, and (d/e) Comparison of the latency of anticipatory muscle activation (AMA) in the flexor carpi radialis (FCR) and extensor carpi radialis (ECR) across the three different trial types between the two groups before and after the intervention. ^**^ *p* < 0.01 and *p < 0.05.

Paired *t*‐tests showed significant improvements in FMA‐UE scores for both groups after the 2‐week intervention (*p* < 0.001). The EA group exhibited greater improvements (from 28.32 to 30.86) compared to the control group (from 31.33 to 32.67), with a significant group effect (*F* = 8.25, *p* = 0.008, *η* ^2^p = 0.221). The EA group also had a higher increase in FMA‐UE scores (2.57 vs. 1.33, *p* = 0.014) (**Figure** **3a**). No changes were observed in MAS scores (*p* > 0.05).

### 3.2 Cortico‐Reticulospinal Changes

#### 3.2.1 SCM+ Incidence

The EA group showed a significant increase in SCM+ incidence post‐intervention (19.03% to 30.30%, *p* = 0.005). No significant change was observed in the control group (*p* > 0.05) (**Figure** **3b**).

#### 3.2.2 Premotor Reaction Time

The EA group exhibited significantly shorter PRT post‐intervention compared to the control group (214.83 ms vs. 261.67 ms, *p* = 0.028). Within‐group comparisons revealed a significant reduction in PRT in the EA group (*p* = 0.007). No change was observed in the control group (*p* > 0.05) (**Figure** **3c**). Two‐way ANOVA with baseline PRT as a covariate showed significant effects of group (*F* = 9.58, *p* = 0.003) and trial type (*F* = 5.24, *p* = 0.009) on PRT. The EA group had significantly shorter PRT than controls in SCM+ (155.70 vs. 220.92 ms; *p* = 0.009) and normal trials (230.75 vs. 292.81 ms; *p* = 0.039) (**Figure** **3c** **)**.

#### 3.2.3 Anticipatory Muscle Activations

For FCR AMAs latency, no significant baseline effect was observed. A two‐way ANOVA revealed a significant group effect for post‐intervention FCR AMAs latency (*F* = 6.31, *p* = 0.015) but no trial type effect (*p* > 0.05) (**Figure** **3d**). Post‐hoc comparisons indicated no significant differences across trial types (*p* > 0.05). Similarly, for ECR AMAs latency, a significant group effect was found (*F* = 4.40, *p* = 0.04), with no significant trial type effects (*p* > 0.05) (**Figure** **3e**).

### 3.3 Cortical Response to Electroacupuncture

#### 3.3.1 Functional Connectivity in SMN and FPN

**FIGURE 4:**
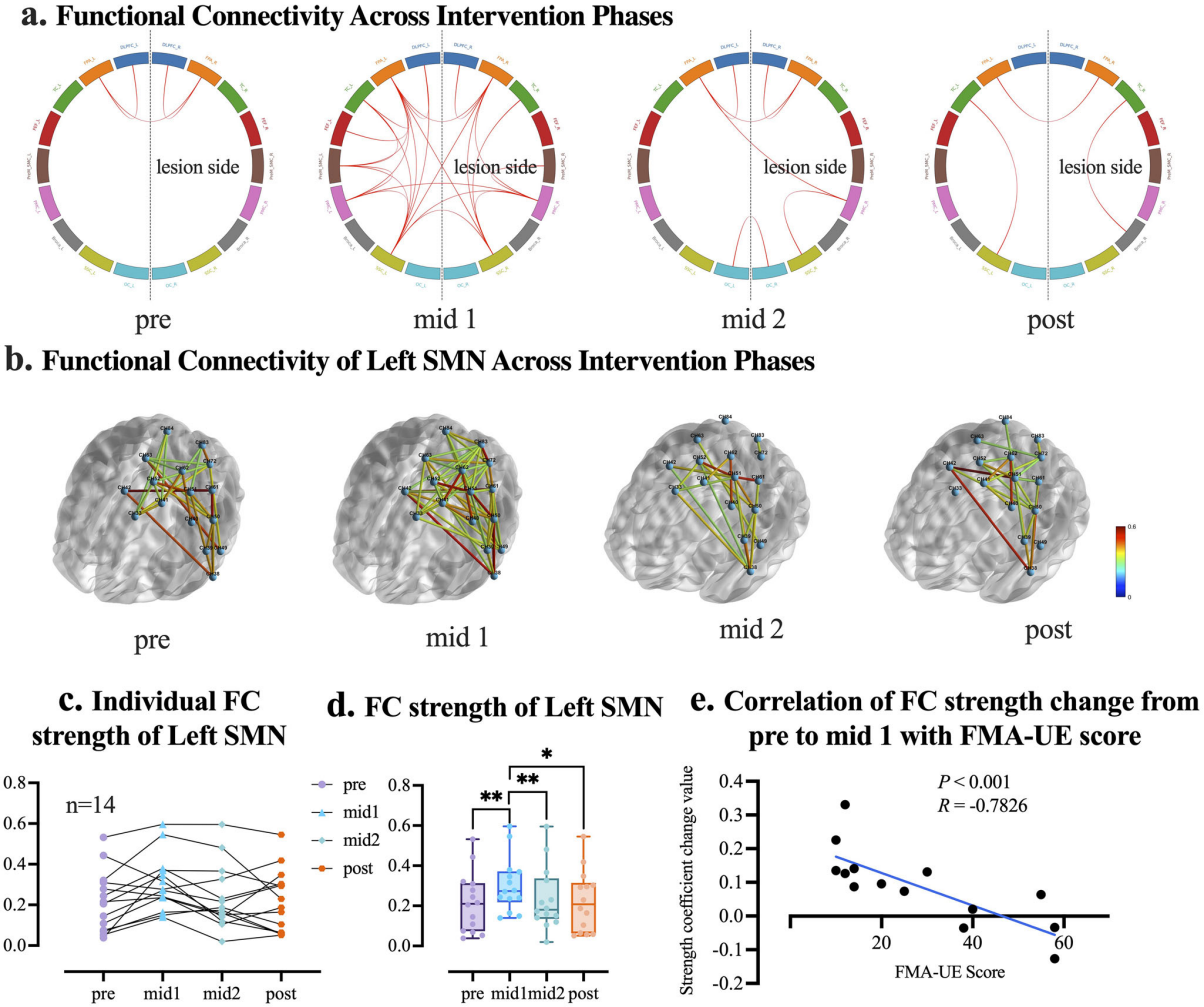
**fNIRS results of brain network analysis across intervention phases. (**a) Functional connectivity of brain areas across the four intervention phases. DLPFC_L and DLPFC_R represent the left and right dorsolateral prefrontal cortex, respectively. The brain areas shown include the frontopolar area (FA), temporal cortex (TC), frontal eye fields (FEF), premotor and supplementary motor cortex (PreM_SMC), primary motor cortex (PMC), Broca's area (Broca), somatosensory cortex (SSC), and occipital cortex (OC), (b) Functional connectivity of the left sensorimotor network (SMN) across the four intervention phases. Channel connections with a Pearson correlation coefficient greater than 0.3 are displayed, with red indicating stronger connectivity, (c) Changes in the functional connectivity of the left sensorimotor cortex at four time points for 14 subjects in the EA group who successfully acquired fNIRS data, (d) Comparison of the average changes in left SMN connection strength for subjects in the EA group at the four time points (pre‐correction results), and (e) Correlation analysis between changes in left sensorimotor network connection strength and initial FMA‐UE scores in the EA group during the early phase of electroacupuncture intervention (from pre to mid 1).

A significant time effect on FC strength in the left SMN was found (*F* = 3.412, *p* = 0.027) (**Figure** **4a**). Post‐hoc comparisons revealed increased FC at mid1 compared to pre (*p* = 0.012), mid2 (*p* = 0.007), and post (*p* = 0.022). After Bonferroni correction, significant differences between mid1 and mid2 persisted (*p* = 0.043). No significant changes were observed for the right SMN and FPN (*p* > 0.05). A significant negative correlation was observed between changes in left SMN FC at mid‐1 and baseline FMA‐UE scores (*p* = 0.001, *R* = −0.783) (**Figure** **4e**).

#### 3.3.2 fALFF Dynamics

One‐way RM‐ANOVA showed a significant time effect on fALFF values in the frontopolar area (*F* = 4.081, *p* = 0.012). Post‐hoc comparisons revealed a decrease in fALFF at post compared to pre and mid1 (*p* < 0.05). No significant changes were found in other brain areas (*p* > 0.05).

## 4 Discussion

This study provides preliminary evidence for the potential clinical benefit of integrating EA with conventional rehabilitation in subacute stroke. Daily EA at LI4 and LI11 acupoints, administered prior to physical therapy, led to significant improvements in upper limb motor function and shortened reaction times. These functional gains were accompanied by objective changes in markers of the CReST‐related pathway modulation, specifically an increased incidence of SE. Furthermore, fNIRS data revealed enhanced functional connectivity within the contralesional sensorimotor network (SMN) during the early phase of the intervention. Together, these findings suggest that EA may promote functional recovery by activating the contralesional hemisphere and optimizing the recruitment of descending motor pathways. As an accessible and cost‐effective intervention, EA offers a practical strategy to address the heavy stroke burden in resource‐limited settings where expensive modalities are unavailable. However, these findings should be interpreted with caution given the limited sample size, short intervention duration, and indirect assessment of CReST plasticity.

### 4.1 CReST in Stroke Recovery and Its Modulation by EA

A key contribution of our study is demonstrating that EA may modulate the CReST, a critical descending motor pathway that is often underutilized in stroke rehabilitation. While recovery has traditionally focused on the integrity of the contralateral corticospinal tract (CST) (Byblow et al. 2015; Lin et al. 2019), the ipsilaterally projecting CReST offers a crucial alternative route for motor adaptation, particularly after severe CST damage (Baker 2011; Paul et al. 2023). However, interventions capable of effectively optimizing this pathway's function remain scarce.

Based on the current understanding of SE, interventions combining acoustic startling stimuli with task‐oriented training (Rahimi and Honeycutt 2020; Swann et al. 2023), magnetic stimulation (Mooney et al. 2023), or peripheral electrical stimulation (Choudhury et al. 2020) have been shown to induce a greater CReST response. In addition, high‐intensity muscle contraction such as handgrip may also be able to induce higher CReST activation (Danielson et al. 2024).

Our findings suggest that EA, as a priming intervention administered prior to physical therapy, represents a novel and accessible approach to modulate CReST‐related activity. Specifically, we observed increased incidence of SCM^+^ and a reduction in PRT at startling trials in the EA group. These changes support the idea that EA at the LI4 and LI11 acupoints may enhance CReST‐related neural plasticity. By facilitating this survival‐oriented neural recruitment, EA potentially contributes to the restoration of motor control, although the present study assessed CReST function indirectly through behavioral and electrophysiological markers.

### 4.2 EA‐Induced Enhancement of Contralateral SMN Connectivity and CReST Recruitment

The enhanced connectivity of the contralesional SMN observed during the early phase of EA intervention may clarify the potential neural mechanism underlying EA‐induced CReST modulation. Promoting information transmission across disconnected networks remains a primary challenge in post‐stroke reorganization (Catani and ffytche 2005; Ganguly et al. 2022). Although the central nervous system, from the cortex to the brainstem and spinal cord, undergoes plastic changes to compensate for motor deficits after stroke (Grefkes and Fink 2011; Karbasforoushan et al. 2019; Urbin 2024), optimizing existing pathways such as the CReST is the most efficient and energy‐conserving strategy. Therefore, the increased excitability of the contralesional SMN likely facilitates signaling along the contralesional descending CReST, thereby serving as a critical neural adaptation.

As shown in **Figure** **2c**, the ascending sensory input generated by EA at the LI4 and LI11 acupoints likely contributed to the enhanced functional connectivity of the contralesional SMN. Although correlation between connectivity and motor improvement does not inherently prove causation, the temporal alignment of these changes specifically during the early intervention phase suggests that EA may act as a neuromodulatory primer. By increasing cortical excitability prior to physical therapy, EA creates a more receptive neural foundation for subsequent training. This distinctive pattern of early‐stage remodeling in the EA group supports the hypothesis that EA drives the neural reorganization necessary for optimized CReST recruitment and functional gains.

### 4.3 Time‐Sensitive Effects of EA on Contralateral SMN Connectivity

During the EA intervention, the enhancement of contralesional SMN connectivity was primarily observed within the first 10 min, indicating a distinct time‐sensitive neuromodulatory effect. The initial enhancement in network connectivity suggests a critical window for neural activation, during which the SMN is most responsive to EA‐induced afferent input. The lack of significant further changes after this period may be attributed to the network reaching an activation plateau or undergoing rapid homeostatic adaptation.

Given that EA in this study serves as a form of peripheral stimulation, these findings align with observations in conventional 1100 Hz neuromuscular electrical stimulations (Carson and Buick 2021), which often show an initial facilitatory phase followed by gradual adaptive weakening. Our observation suggests that the duration and intensity of EA may differentially impact network excitability, with the most pronounced modulation occurring in the early phase. Therefore, future protocols integrating EA with physical therapy should prioritize optimizing the priming duration and intensity to maximize CReST regulation and subsequent motor recovery.

### 4.4 Limitations

Several limitations should be considered when interpreting these findings. First, as an exploratory study, this work provides preliminary evidence for EA‐induced CReST modulation, a relationship not yet conclusively established. The relatively small sample size and the absence of a sham‐controlled group necessitate caution when making causal inferences. Furthermore, as the study focused on subacute stroke patients, the findings may not be generalizable to patients in chronic recovery stages. Future studies involving larger cohorts, sham‐controlled designs, and diverse patient populations across different recovery stages are needed to validate and extend these findings. Second, the intervention duration was limited to two weeks, leaving the long‐term effects of EA on neural plasticity and motor recovery unclear. The lack of extended follow‐up prevents conclusions regarding the durability of the observed functional gains. To fully understand the therapeutic potential of this approach, future research should examine the impact of longer‐term EA interventions. Last, while fNIRS provided valuable insights into cortical connectivity, it has inherent limitations in spatial resolution and depth penetration. The CReST function was inferred from behavioral and electrophysiological markers rather than directly measured using structural or tract‐specific neuroimaging techniques. Integrating fNIRS with higher‐resolution techniques, such as functional magnetic resonance imaging or diffusion tensor imaging, would provide more detailed evidence of the neural mechanisms underlying EA‐induced CReST recruitment.

## 5 Conclusions

In conclusion, this study provides preliminary evidence that EA may enhance motor recovery in subacute stroke patients, potentially through modulation of CReST‐related pathways and activation of the contralesional SMN. Our findings suggest that EA acts as a neuromodulatory primer, with the observed enhancement of contralesional SMN activity indicating a time‐sensitive window for neural responsiveness. These results highlight the potential of EA to optimize the neural foundation for subsequent rehabilitation training. However, given the methodological limitations, including the small sample size, the absence of a sham‐controlled design, and the indirect assessment of CReST function, these findings should be interpreted with caution. Further large‐scale, well‐controlled studies are warranted to confirm these effects and more definitely clarify the underlying neural mechanisms of EA‐induced CReST recruitment.

## Author Contributions

**Tangzhu Yang**: data curation. **Zhenhong Li**: methodology, software, formal analysis. **Nan Xia**: conceptualization, writing – review and editing, writing – original draft. **Chaoyuan Liang**: methodology, software, formal analysis. **Xiaohua Han**: conceptualization, writing – review and editing, supervision. **Jinghong Li**: data curation. **Mingzhu Wang**: data curation. **Qiongfang Wu**: conceptualization, writing – original draft. **Zejian Chen**: data curation. **Minghui Gu**: data curation. **Yong Chen**: conceptualization, supervision, funding acquisition, project administration.

## Funding

This project was supported in part by the Hubei Provincial Major Science and Technology Special Project (No. 2023BCA002).

## Ethics Statement

The study was approved by the Medical Ethics Committee of Tongji Hospital (Approval No. TJ‐IRB20230407).

## Consent

Written informed consents were obtained from all individual participants prior to enrollment in the study.

## Conflicts of Interest

The authors declare no conflicts of interest.

## Clinical Trial Registration

The study was registered in the Chinese Clinical Trial Registry (Registration No. ChiCTR2200066858; http://www.chictr.org.cn/) on December 20, 2022.

## Supporting information




**Supplementary Materials**: brb371496‐sup‐0001‐SuppMat.xlsx

## Data Availability

The clinical data, fNIRS, and sEMG data collected during this study are not publicly available but may be obtained from the corresponding authors upon reasonable request.
